# Curcumin modulates dopaminergic receptor, CREB and phospholipase c gene expression in the cerebral cortex and cerebellum of streptozotocin induced diabetic rats

**DOI:** 10.1186/1423-0127-17-43

**Published:** 2010-05-31

**Authors:** T Peeyush Kumar, Sherin Antony, G Gireesh, Naijil George, CS Paulose

**Affiliations:** 1 Molecular Neurobiology and Cell Biology Unit, Centre for Neuroscience, Cochin University of Science and Technology, Cochin- 682 022, Kerala, India

## Abstract

Curcumin, an active principle component in rhizome of *Curcuma longa*, has proved its merit for diabetes through its anti-oxidative and anti-inflammatory properties. This study aims at evaluating the effect of curcumin in modulating the altered dopaminergic receptors, CREB and phospholipase C in the cerebral cortex and cerebellum of STZ induced diabetic rats. Radioreceptor binding assays and gene expression was done in the cerebral cortex and cerebellum of male Wistar rats using specific ligands and probes. Total dopaminergic receptor binding parameter, B_max _showed an increase in cerebral cortex and decrease in the cerebellum of diabetic rats. Gene expression studies using real time PCR showed an increased expression of dopamine D1 and D2 receptor in the cerebral cortex of diabetic rats. In cerebellum dopamine D1 receptor was down regulated and D2 receptor showed an up regulation. Transcription factor CREB and phospholipase C showed a significant down regulation in cerebral cortex and cerebellum of diabetic rats. We report that curcumin supplementation reduces diabetes induced alteration of dopamine D1, D2 receptors, transcription factor CREB and phospholipase C to near control. Our results indicate that curcumin has a potential to regulate diabetes induced malfunctions of dopaminergic signalling, CREB and Phospholipase C expression in cerebral cortex and cerebellum and thereby improving the cognitive and emotional functions associated with these regions. Furthermore, in line with these studies an interaction between curcumin and dopaminergic receptors, CREB and phospholipase C is suggested, which attenuates the cortical and cerebellar dysfunction in diabetes. These results suggest that curcumin holds promise as an agent to prevent or treat CNS complications in diabetes.

## Introduction

Diabetes mellitus is a heterogeneous disease characterized by chronic hyperglycaemia and requires long-term management. Chronic changes in the antecedent level of glycaemia induce alterations in brain glucose metabolism in rodents [1,2]. Chronic hyperglycemia in diabetes can lead to various complications, affecting the CNS [3]. A continuous systemic supply of glucose is essential for normal cerebral metabolism [4].

Controlling blood sugar is essential for avoiding long-term complications of diabetes like learning and memory. Although mechanisms leading to cortical and cerebellar dysfunction associated with diabetic complications are not completely understood, brain cells are particularly vulnerable to oxidative stress [5]. Oxidative stress, leading to an increased production of reactive oxygen species, as well as lipid peroxidation is increased in diabetes [6-8]. Hyperglycemia causes the autoxidation of glucose, glycation of proteins, and the activation of polyol metabolism [9]. These changes accelerate the generation of reactive oxygen species to increase oxidative modifications of lipids, DNA, and proteins in various tissues. Oxidative stress is believed to play an important role in the development of complications in diabetes associated neuronal disorders [9]. Greater understanding of CNS (CNS) involvement could lead to new strategies to prevent or reverse the damage caused by diabetes mellitus.

Antioxidant agents from diet have a significant therapeutic influence on various neurodegenerative disorders associated with diabetes and oxidative stress [10,11]. Curcuminoids, the main components in Curcuma species, share a common unsaturated alkyl-linked biphenyl structural feature and are responsible for their major pharmacological effects. The biological and chemical properties of curcuminoids were reported [12] A number of experimental studies have demonstrated CUR's antioxidant and neuroprotective potential [13,14] Also curcumin modulates the expression of various molecular targets, such as transcription factors, enzymes, cytokines, cell cycle proteins, receptors and adhesion molecules [15]. Diabetes mellitus has also been reported to be accompanied by behavioural and reduced motor activity [16]. One unifying mechanism which lies behind this neuronal injury is the excessive free radical generation from the auto oxidation of elevated intracellular glucose levels. Curcumin may antagonise the deficit of glucose energy metabolism or oxidative stress related to cognitive impairment associated with diabetes.

Diabetes is also found to be associated with changes in somatic sensations which involve the cerebellum, cerebral cortex and thalamus. Symptoms, like loss of pain, impaired touch perception and decreased position sense, have been commonly documented in a diabetic patient [17]. Dopamine in the CNS is involved in the control of both motor and emotional behavior [18] and peripherally modulates insulin secretion in the pancreatic islets [19]. Nafadotride, a preferential antagonist of dopamine D3 receptors administered at low doses directly into the cerebellum, has been shown to activate locomotor activity [20]. The secretion of insulin by β-cells of the endocrine pancreas is regulated by glucose and other circulating nutrients. It is also modulated by several hormones and neurotransmitters, among which dopamine plays a prominent role.

CREB is a protein that is a transcription factor. It binds to certain DNA sequences called cAMP response elements and, thereby, increases or decreases the transcription of the downstream genes [21]. Genes whose transcription is regulated by CREB include: c-fos, BDNF (Brain-derived neurotrophic factor), tyrosine hydroxylase and neuropeptides such as somatostatin, enkephalin, VGF and corticotropin-releasing hormone [21]. In neuronal tissue, CREB regulation by nerve growth factor and insulin-like growth factor-1 is essential for neuronal plasticity, full axonal development, memory consolidation, and neuroprotection [22,23]. The Phospholipase C activity decline in the brain is expected to affect mainly the 18:0/20:4 molecular species of DAG because this is the principal molecular species of phosphoinositides in the nervous tissue [24].

Hyperglycaemia is associated with a number of physiological changes, the most profound effects are seen in the brain, where glucose is the major substrate for energy metabolism and both local energy store and the supply of alternate sources are limited. The initiating events in hyperglycemic encephalopathy still are not understood completely. But brain injury appears to result from a number of processes that are initiated when blood glucose concentration is altered. However, the action mechanisms of this remain obscure. Therefore, this study was designed to investigate the beneficial effect of curcumin a neuroprotective agent, on impairment in dopaminergic receptors, CREB and phospholipase C in the cerebral cortex and cerebellum of STZ-induced diabetic rats. Our present study on curcumin dependent regulation of dopaminergic receptors, transcription factor CREB and phosphor lipase C amelioration of cortical and cerebellar cells will certainly enlighten novel therapeutic possibilities in diabetes treatment.

## Materials and methods

Bio chemicals used in the present study were purchased from Sigma Chemical Co., St. Louis, USA. All other reagents of analytical grade were purchased locally. [^3^H] Dopamine were purchased from NEN Life Sciences Products Inc., Boston, U.S.A. dopamine and curcumin were from Sigma Chemical Co., USA. Tri-reagent kit was purchased from MRC, USA. Real Time PCR Taqman probe assays on demand were from Applied Biosystems, Foster City, CA, USA.

Male adult Wistar rats of 180-240 g body weight were used for all experiments. The animals were allowed to acclimatise for 2 weeks before the experiment. They were housed individually in separate cages under 12 hour light and 12 hour dark periods. Rats had free access to standard food and water ad libitum. All animal care and procedures were done in accordance with the Institutional and National Institute of Health guidelines. All efforts were made to minimize the number of animals used and their suffering. Diabetes was induced in rats by single intra femoral vein injection of STZ freshly dissolved in 0.1 M citrate buffer, pH 4.5, under anaesthesia [25]. STZ was given at a dose of 55 mg/kg body weight [26,27]. Animals were divided into the following groups: I) Control ii) diabetic iii) insulin-treated diabetic iv) curcumin-treated diabetic rats. Each group consisted of 6-8 animals. The insulin-treated diabetic group received subcutaneous injections (1 Unit/kg body weight) of Lente and Plain insulin (Boots India) daily during the entire period of the experiment. The last injection was given 24 hours before sacrificing the rats. Curcumin treated groups received 60 mg/kg suspension of curcumin orally [28] for the entire period of the experiment. Curcumin was suspended in 0.5% w/v sodium carboxymethylcellulose immediately before administration in constant volume of 5 ml/kg body weight. Rats were sacrificed on 15th day by decapitation. The cerebellum was dissected out quickly over ice according to the procedure of Glowinski and Iversen, 1966 [29] and the tissues collected were stored at -80°C until assayed.

### Estimation of blood glucose

Blood glucose was estimated by the spectrophotometer method using glucose oxidase-peroxidase reactions. Blood samples were collected from the tail vein at 0 hour (Before the start of the experiment), 3rd, 6th, 10th and 14th day and the glucose levels were estimated subsequently. Along with this blood samples were collected 3 hrs after the administration of morning dose of insulin and curcumin. The results were expressed in terms of milligram per decilitre of blood.

### Total Dopamine receptor binding studies in the cerebellum

DA receptor assay was done using [^3^H]DA according to Madras *et al*., 1988 [30]. Cerebellum was homogenised in a polytron homogeniser with 20 volumes of cold 50 mM Tris-HCl buffer, along with 1 mM EDTA, 0.01%ascorbic acid, 4 mM MgCl_2_, 1.5 mM CaCl_2_, pH. 7.4 and centrifuged at 38,000 × g for 30 min at 4°C. The pellet was washed twice by rehomogenization and centrifuged twice at 38,000 × g for 30 min at 4°C. This was resuspended in appropriate volume of the buffer containing the above mentioned composition.

Binding assays were done using different concentrations i.e., 0.25 nM-1.5 nM of [^3^H]DA in 50 mM Tris-HCl buffer, along with 1 mM EDTA, 0.01% ascorbic acid, 1 mM MgCl_2_, 2 mM CaCl_2_, 120 mM NaCl, 5 mM KCl, pH.7.4 in a total incubation volume of 250 μl containing 200-300 μg of proteins. Specific binding was determined using 100 μM unlabelled dopamine.

Tubes were incubated at 25°C for 60 min. and filtered rapidly through GF/B filters (Whatman). The filters were washed quickly by three successive washing with 5.0 ml of ice cold 50 mM Tris buffer, pH 7.4. Bound radioactivity was counted with cocktail-T in a Wallac 1409 liquid scintillation counter. The non-specific binding determined showed 10% in all our experiments.

### Protein determination

The amount of protein was measured by the method of Lowry et al., 1951 [31] using bovine serum albumin as standard. The intensity of the purple blue colour formed was proportional to the amount of protein, which was read in a spectrophotometer at 660 nm.

### Receptor data analysis

The receptor binding parameters were determined using Scatchard analysis [32]. The specific binding was determined by subtracting non-specific binding from the total. The binding parameters, maximal binding (B_max_) and equilibrium dissociation constant (K_d_), were derived by linear regression analysis by plotting the specific binding of the radioligand on X-axis and bound/free on Y-axis using Sigma plot software (version 2.0, Jandel GmbH, Erkrath, Germany). The maximal binding is a measure of the total number of receptors present in the tissue and the equilibrium dissociation constant is the measure of the affinity of the receptors for the radioligand. The K_d _is inversely related to receptor affinity.

### Analysis of gene expression by Real-Time PCR

RNA was isolated from the cerebellum of experimental rats using the Tri-reagent (MRC, USA). Total cDNA synthesis was performed using ABI PRISM cDNA archive kit in 0.2 ml microfuge tubes. The reaction mixture of 20 μl contained 0.2 μg total RNA, 10 × RT buffer, 25 × dNTP mixture, 10 × random primers, MultiScribe RT (50 U/μl) and RNase free water. The cDNA synthesis reactions were carried out at 25°C for 10 minutes and 37°C for 2 hours using an Eppendorf Personal Cycler. Real-time PCR assays were performed in 96-well plates in ABI 7300 real-time PCR instrument (Applied Biosystems). The primers and probes were purchased from Applied Biosystems, Foster City, CA, USA. The TaqMan reaction mixture of 20 μl contained 25 ng of total RNA-derived cDNAs, 200 nM each of the forward primer, reverse primer and TaqMan probe for assay on demand and endogenous control β-actin and 12.5 μl of Taqman 2× Universal PCR Master Mix (Applied Biosystems) and the volume was made up with RNAse free water. The following thermal cycling profile was used (40 cycles): 50°C for 2 min, 95°C for 10 min, 95°C for 15 sec and 60°C for 1 min.

Fluorescence signals measured during amplification were considered positive if the fluorescence intensity was 20-fold greater than the standard deviation of the baseline fluorescence. The ^ΔΔ^CT method of relative quantification was used to determine the fold change in expression. This was done by normalizing the resulting threshold cycle (CT) values of the target mRNAs to the CT values of the internal control β-actin in the same samples (^Δ^CT = CT_Target _- CT _β-actin_). It was further normalized with the control (^ΔΔ^CT = ^Δ^CT - CT_Control_). The fold change in expression was then obtained as (2^-^^ΔΔ^C T) and the graph was plotted using log 2^-^^ΔΔ^CT.

### Statistics

Statistical evaluations were done by ANOVA, expressed as mean ± S.E.M using In Stat (Ver.2.04a) computer programme.

## Results

Blood glucose level of all rats before STZ administration was within the normal range. STZ administration led to a significant increase (p < 0.001) in blood glucose level of diabetic rats compared to control rats. Insulin and Curcumin treatment were able to significantly reduce (p < 0.001) the increased blood glucose level to near the control value compared to diabetic group (Table 1).

**Table 1:** Blood glucose (mg/dl) level in Experimental rats

Animal status	0 day (Before STZ injection)	3^rd ^day (Initial)	6^th ^day	10^th ^day	14^th ^day (Final)
Control	87.2 ± 1.4	86.6 ± 1.2	83.2 ± 1.2	86.3 ± 1.2	85.7 ± 1.5

Diabetic	85.3 ± 1.3	257.3 ± 0.9	318.2 ± 1.6	307.8 ± 1.3	320.5 ± 1.3***

D + I	86.4 ± 0.9	249.8 ± 1.2	303.6 ± 0.8	185.9 ± 1.5	137.0 ± 1.3^ψψψ ϕϕϕ^

D+C	89.3 ± 1.5	259.7 ± 1.8	305 ± 0.9	190 ± 1.7	175.6 ± 1.0^ψψψ ϕϕϕ^

Values are mean ± S.E.M of 4-6 rats in each group. Each group consist of 6-8 rats

*** P < 0.001 when compared to control, ^ψψψ^P < 0.001 when compared to diabetic group, ^ϕϕϕ ^p < 0.001 when compared with initial reading

D + I- Insulin treated diabetic rats

D+C- Curcumin treated diabetic rats

### Total dopamine receptor analysis

#### a) Scatchard analysis of [^3^H] dopamine binding against dopamine in the cerebral cortex and cerebellum of control and experimental rats

The Scatchard analysis showed that the B_max _and K_d _of the [^3^H] dopamine receptor binding decreased significantly (p < 0.001) in the cerebral cortex of diabetic rats compared to control group. In Curcumin and insulin treated diabetic groups, B_max _reversed to near control value. K_d _of insulin treated and Curcumin group reversed to near control. (Table 2, 3)

**Table 2:** Scatchard analysis of [^3^H] dopamine binding against dopamine in the cerebral cortex of control, and experimental rats

Animal status	B_max _(fmoles/mg protein)	K_d _(nM)
Control	23 ± 3.7	2.01 ± 0.05

Diabetic	67 ± 5.7***	6.17 ± 0.06**

D + I	19.8 ± 4.1^ψψψ^	2.4 ± 0.05^ψψ^

D + C	21.7 ± 6.6^ψψψ^	1.96 ± 0.06^ψψ^

Values are mean ± S.E.M of 4-6 separate experiments. Each group consist of 6-8 rats *** P < 0.001 when compared to control, ^ψψψ^P < 0.001 when compared to diabetic group **P < 0.01 when compared to control group ^ψψ ^P < 0.01 when compared to diabetic group.

D + I- Insulin treated diabetic rats

D+C- Curcumin treated diabetic rats

**Table 3:** Scatchard analysis of [^3^H] dopamine binding against dopamine in the cerebellum of control, and experimental rats

Animal status	B_max _(fmoles/mg protein)	K_d _(nM)
Control	112 ± 5.4	3.8 ± 0.14
Diabetic	22 ± 3.6***	2.3 ± 0.05**

D + I	116 ± 4.3^ψψψ^	3.2 ± 0.13^@@^

D + C	91.1 ± 3.87^ψψψ^	4.0 ± 0.03^@@^

Values are mean ± S.E.M of 4-6 separate experiments. Each group consist of 6-8 rats ***P < 0.001 when compared to control, ^ψψψ^P < 0.001 when compared to diabetic group **P < 0.01 when compared to control group ^@@^P < 0.01 when compared to diabetic group.

D + I- Insulin treated diabetic rats

D+C- Curcumin treated diabetic rats

#### Real Time-PCR Analysis of dopamine D1 Receptor in cerebral cortex and cerebellum of control and experimental rats

**Figure 1:**
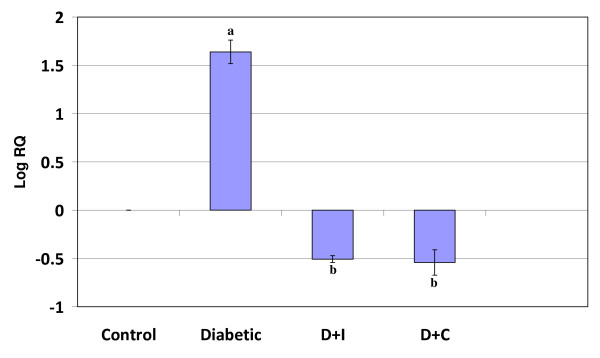
**Real Time PCR amplification of dopamine D1 receptor mRNA from the cerebral cortex of control and experimental rats**. Values are mean ± S.D of 4-6 separate experiments. Each group consist of 6-8 rats Relative Quantification values and standard deviations are shown in the table. The relative ratios of mRNA levels were calculated using the ^ΔΔ^CT method normalized with β-actin CT value as the internal control and Control CT value as the calibrator. **a **p < 0.001 when compared with control, **b **p < 0.001 when compared with diabetic group. D+I - Insulin treated diabetic group. D+C- Curcumin treated diabetic group.

**Figure 2:**
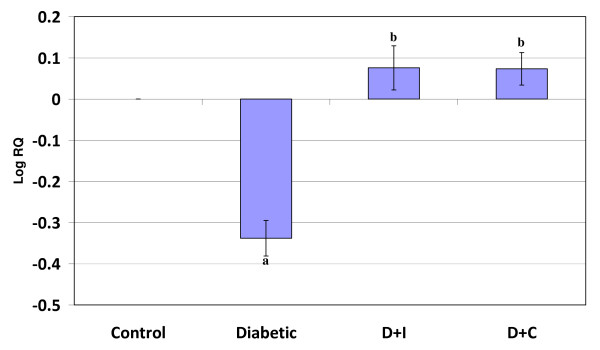
**Real Time PCR amplification of dopamine D1 mRNA from the cerebellum of control and experimental rats**. Values are mean ± S.D of 4-6 separate experiments. Each group consist of 6-8 rats Relative Quantification values and standard deviations are shown in the table. The relative ratios of mRNA levels were calculated using the ^ΔΔ^CT method normalized with β-actin CT value as the internal control and Control CT value as the calibrator. **a **p < 0.001 when compared with control **b **p < 0.001 when compared with diabetic group. D+I - Insulin treated diabetic group. D+C- Curcumin treated diabetic group.

Real Time-PCR analysis showed that the dopamine D1 receptor gene expression was significantly increased (p < 0.001) in the cerebral cortex and decreased (p < 0.001) cerebellum in diabetic condition. Insulin and curcumin treatment reversed the altered expression to near control (Figure 1 and 2).

#### Real Time-PCR Analysis of dopamine D2 Receptor in cerebral cortex and cerebellum of control and experimental rats

**Figure 3:**
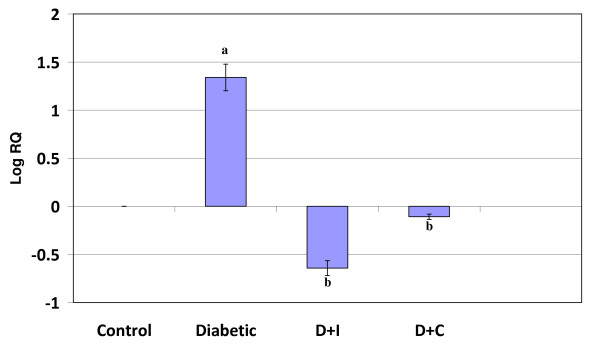
**Real Time PCR amplification of dopamine D2 mRNA from the cerebral cortex of control and experimental rats**. Values are mean ± S.D of 4-6 separate experiments. Each group consist of 6-8 rats Relative Quantification values and standard deviations are shown in the table. The relative ratios of mRNA levels were calculated using the ^ΔΔ^CT method normalized with β-actin CT value as the internal control and Control CT value as the calibrator. **a **p < 0.001 when compared with control **b **p < 0.001 when compared with diabetic group. D+I - Insulin treated diabetic group. D+C- Curcumin treated diabetic group.

**Figure 4:**
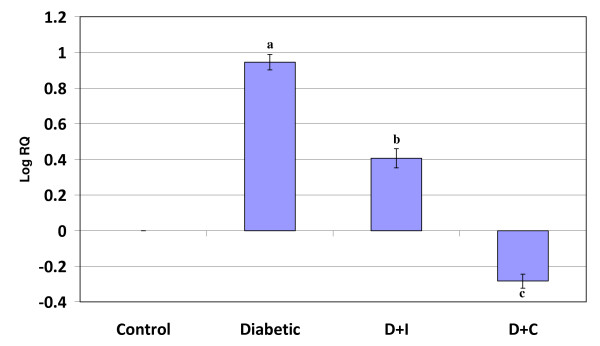
**Real Time PCR amplification of dopamine D2 mRNA from the cerebellum of control and experimental rats**. Values are mean ± S.D of 4-6 separate experiments. Each group consist of 6-8 rats Relative Quantification values and standard deviations are shown in the table. The relative ratios of mRNA levels were calculated using the ^ΔΔ^CT method normalized with β-actin CT value as the internal control and Control CT value as the calibrator. **a **p < 0.001 when compared with control **b **p < 0.01 when compared with diabetic group **c **p < 0.001 when compared with diabetic group. D+I - Insulin treated diabetic group. D+C- Curcumin treated diabetic group.

Real Time-PCR analysis showed that the dopamine D2 receptor gene expression in the cerebral cortex and cerebellum was significantly increased (p < 0.001) in diabetic condition and it reversed to near control value in insulin and curcumin treated diabetic rats (Figure 3 and 4).

#### Real Time-PCR Analysis of CREB in the cerebral cortex and cerebellum of control and experimental rats

**Figure 5:**
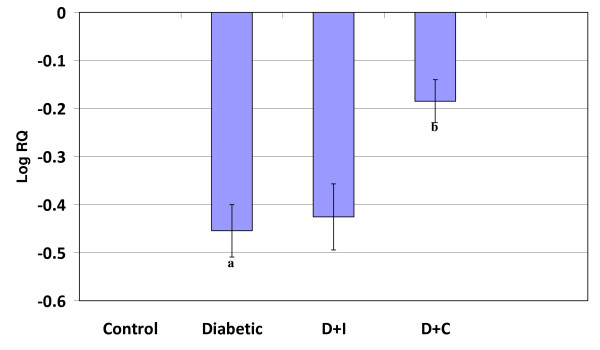
**Real Time PCR amplification of CREB mRNA from the cerebral cortex of control and experimental rats**. Values are mean ± S.D of 4-6 separate experiments. Each group consist of 6-8 rats Relative Quantification values and standard deviations are shown in the table. The relative ratios of mRNA levels were calculated using the ^ΔΔ^CT method normalized with β-actin CT value as the internal control and Control CT value as the calibrator. **a **p < 0.001 when compared with control **b **p < 0.01 when compared with diabetic group. D+I - Insulin treated diabetic group. D+C- Curcumin treated diabetic group.

**Figure 6:**
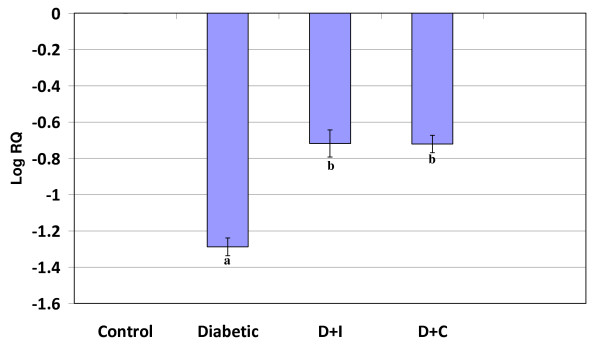
**Real Time PCR amplification of CREB mRNA from the cerebellum of control and experimental rats**. Values are mean ± S.D of 4-6 separate experiments. Each group consist of 6-8 rats Relative Quantification values and standard deviations are shown in the table. The relative ratios of mRNA levels were calculated using the ^ΔΔ^CT method normalized with β-actin CT value as the internal control and Control CT value as the calibrator. **a **p < 0.001 when compared with control **b **p < 0.01 when compared with diabetic group. D+I - Insulin treated diabetic group. D+C- Curcumin treated diabetic group.

Real Time-PCR analysis showed that the CREB gene expression in the cerebral cortex and cerebellum was significantly decreased (p < 0.001) in diabetic condition. In cerebral cortex, curcumin treatment reversed the altered expression to near control while insulin treatment shows no significant reversal. In cerebellum curcumin and insulin treatment reversed the altered expression to near control value (Figure 5 and 6).

#### Real Time-PCR Analysis of phospholipase C in the cerebral cortex and cerebellum of control and experimental rats

**Figure 7:**
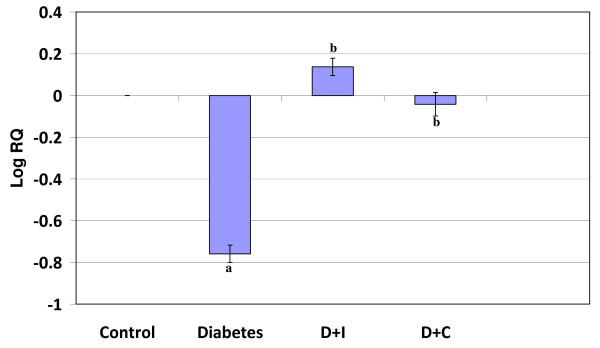
**Real Time PCR amplification of phospholipase C mRNA from the cerebral cortex of control and experimental rats**. Values are mean ± S.D of 4-6 separate experiments. Each group consist of 6-8 rats Relative Quantification values and standard deviations are shown in the table. The relative ratios of mRNA levels were calculated using the ^ΔΔ^CT method normalized with β-actin CT value as the internal control and Control CT value as the calibrator. **a **p < 0.001 when compared with control **b **p < 0.001 when compared with diabetic group. D+I - Insulin treated diabetic group. D+C- Curcumin treated diabetic group.

**Figure 8:**
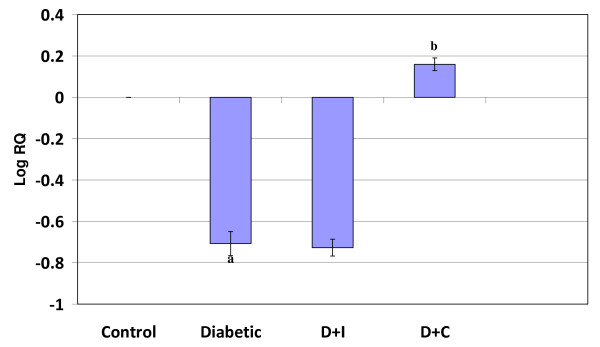
**Real Time PCR amplification of phospholipase C mRNA from the cerebellumof control and experimental rats**. Values are mean ± S.D of 4-6 separate experiments. Each group consist of 6-8 rats Relative Quantification values and standard deviations are shown in the table. The relative ratios of mRNA levels were calculated using the ^ΔΔ^CT method normalized with β-actin CT value as the internal control and Control CT value as the calibrator. **a **p < 0.001 when compared with control **b **p < 0.001 when compared with diabetic group. D+I - Insulin treated diabetic group. D+C- Curcumin treated diabetic group.

Real Time-PCR analysis showed that the phospholipase C gene expression in the cerebral cortex and cerebellum was significantly decreased (p < 0.001) in diabetic condition. In cerebral cortex curcumin and insulin treatment reversed the altered expression in diabetes to near control. In cerebellum curcumin treatment reversed the altered expression to near control while insulin treatment shows no significant reversal (Figure 7 and 8).

## Discussion

There is a complex relationship among diabetes mellitus and CNS, the present study is an attempt to investigate the role of curcumin in regularising the altered dopaminergic and second messenger expression in the cerebral cortex and cerebellum of STZ-induced diabetic rats. Diabetic encephalopathy, characterized by impaired cognitive functions and neurochemical and structural abnormalities, may involve direct neuronal damage. Therefore, we have assessed the possibility of curcumin supplementation that target oxidative stress which would help in preventing and/or delaying the progression of diabetes and associated neuronal injury in cerebral cortex and cerebellum. This study demonstrated for the first time that STZ-induced diabetes produces a marked attenuation of cerebral cortical and cerebellum function mediated through dopaminergic receptors, phospholipase C activity and transcription factor CREB in the Wistar rats.

The STZ diabetic rat serves as an excellent model to study the molecular, cellular and morphological changes in brain induced by stress during diabetes [33]. In the present study, STZ-induced rats were used as an experimental model for diabetes, since they provides a relevant example of endogenous chronic oxidative stress due to the resulting hyperglycemia [34]. The facts' that increased blood glucose level and decreased body weight, observed during diabetes, are similar with previous reports as a result of the marked destruction of insulin secreting pancreatic β-cells by STZ [25]. Previous reports showed that curcumin has the potential to protect pancreatic islet cells against STZ-induced death and dysfunction [35] and increase plasma insulin level in diabetic mice [36]. The results of this study have demonstrated that insulin and curcumin treatment to STZ-induced diabetic rats can have beneficial effects in reducing blood glucose levels to near control. The central complications of hyperglycemia also include the potentiation of neuronal damage observed following hypoxic/ischemic events, as well as stroke. Glucose utilization is decreased in the brain during diabetes [37], providing a potential mechanism for increased vulnerability to acute pathological events.

Dopamine is the predominant catecholamine neurotransmitter in the mammalian brain, where it controls a variety of functions including locomotor activity, cognition, emotion, positive reinforcement, food intake, and endocrine regulation. This catecholamine also plays multiple roles in the periphery as a modulator of cardiovascular function, catecholamine release, hormone secretion, vascular tone, renal function, and gastrointestinal motility [38]. Dopamine receptors are reported to be increased in diabetes causing significant alterations in central dopaminergic system [39]. It is hypothesized that the cerebral cortex participates in the memory, attention, perceptual awareness, thought, language, and consciousness which are necessary for the normal life style. In the present study the scatchard analysis of total dopamine receptors in diabetic rats showed an increased receptor binding or number in cerebral cortex when compared to control, thus contributing to neurological dysfunctions associated with cortex. Earlier reports showed significant alterations in neurotransmitters during hyperglycaemia and causes degenerative changes in neurons of the CNS [40]. A converse pattern of the modulation of total dopaminergic receptors was obtained in cerebellum, which is responsible for the coordination of voluntary motor movement, balance, equilibrium and declarative memory. Total dopamine receptor density was decreased in the cerebellum of diabetic rats when compared to control indicating an unbalance in dopaminergic neural transmission. Furthermore, many behavioural studies have shown evidence that the dopamine system plays an important role in regulating exploratory and locomotor behavior [41,42]. The current data reveal a significant reversal of this altered binding parameter to near control in curcumin and insulin treatment. Thus we speculated that curcumin has an ability to modulate dopaminergic receptors there by ameliorating the impaired cortical performance associated with diabetes. Diabetes mellitus has been reported to be accompanied by a number of behavioural and hormonal abnormalities, including reduced locomotor activity [43]. The present experiments further revealed the effect of curcumin to modulate the dopaminergic receptors in the cerebellum by standardising the altered expression to a normal level.

DA D_1 _receptors are highly expressed in basal ganglia followed by cerebral cortex, hypothalamus and thalamus. The gene expression studies of dopamine D1 receptors showed an increase in the cortex of diabetic rats which confirm and extend our observations of total dopamine receptors. Dopamine D1 receptor seems to mediate important actions of dopamine to control movement, cognitive function and cardiovascular function. The DA D_1 _receptors in the brain are linked to episodic memory, emotion, and cognition. Diabetes mellitus has been reported to cause degenerative changes in neurons of the CNS [44,45,40]. Our study showed that diabetes can regulate the expression of dopamine D1 receptor which may reduce the central cortical function. Furthermore, curcumin and insulin exhibited a tendency for decreasing this altered mRNA expression to near control. Such interference with the dopaminergic system could explain, at least in part, the ameliorative effect of curcumin on CNS. In agreeable with the total dopamine receptor change in the cerebellum dopamine D1 receptor expression was down regulated in the diabetic rats when compared to control. Haloperidol and SCH23390, a selective dopamine D1 receptor antagonist, significantly reduced spontaneous locomotor activity in diabetic mice, but not in non diabetic mice [46]. In our study, curcumin and insulin increased the dopamine D1 receptor expression levels in the cerebellum, which suggests that the curcumin supplementation influenced the functional regulation of these receptors to maintain normal dopaminergic function and this might also be involved as a mechanism of preventing cerebellar dysfunctions.

The interest in learning dopamine D2 receptor expression begins with the hypothesis that dopamine D2 receptors are involved in the pathophysiology of schizophrenia and in the mechanism of antipsychotic drug action [47]. Also widespread distribution of dopamine D2 receptors in the cerebral cortex is of considerable clinical significance because this may be the site for regulation of cognitive deficits [48]. Thus, our findings should bring attention to the cortex as a possible site of dysfunction in diseases like diabetes mellitus. To examine whether dopamine D2 receptors are altered in diabetes, we examined the expression levels of D2 in the cortex, and the cerebellum, because these tissues are regions to which dopaminergic neurons project, and are well known to be related to memory, attention, perceptual awareness, thought, language, consciousness and motor function. The present study showed that dopamine D2 receptors expression of cortex and cerebellum in diabetic rats where up regulated when compared to control. These results may indicate an alteration of the dopamine system in diabetes, because it is well known that dopamine is a principal modulator of higher functions including attention working memory [49] and motor control [50]. The increase in the central dopaminergic postsynaptic receptors has been related to decrease the locomotor and ambulatory activity in STZ-induced diabetic rats [51,52]. It was reported that injection of dopamine D2 agonist into lobules 9 and 10 of the cerebellar cortex, induced balance and motor coordination disturbances in the rotarod test [53]. It has been suggested that curcumin reverses the effects of diabetes on dopamine D2 receptors in the cortex and cerebellum to near control level.

Previous studies from our lab have established the role of neurotransmitters in maintaining the glucose homeostasis [54-57]. Thus it is evident that the various neurotransmitter systems, including - Dopamine, acetylcholine, glutamate, GABA; are modulated by diabetes. The coordinated activation and inhibition of different neurotransmitter systems in control rats are disrupted during diabetes. The synergistic effect of neurotransmitters receptor alterations results in CNS disorders during diabetes. Puglisi et al (1995) [58], reported the regulatory role dopamine D1 and D2 receptors in modulating acetylcholine activity. Also hippocampal D2 receptors modulate spatial working memory functions, and this effect is due to the increased acetylcholine release associated with D2 receptor stimulation [59,60].

The cAMP response element-binding protein (CREB) plays a pivotal role in dopamine receptor-mediated nuclear signaling and neuroplasticity [61]. Here we demonstrate the significance of CREB gene expression in the cerebral cortex and cerebellum of STZ-induced diabetes rats. Our findings showed a significant down regulation of CREB in cerebral cortex and cerebellum of diabetic rats, when compared to control. The study of the dopamine receptors expression in relation with CREB phosphorylation in diabetes is an important step toward elucidating the relationship between molecular adaptations and behavioural consequences. CREB proteins in neurons are thought to be involved in the formation of long-term memories; this has been shown in the marine snail Aplysia, the fruit fly Drosophila melanogaster, and in rats. CREB is necessary for the late stage of long-term potentiation. CREB also has an important role in the development of drug addiction [62]. It is therefore important to identify the elements that modulate dopaminergic receptor expressions and phosphorylation of CREB and there by its expression in the nucleus. Drugs that stimulate dopamine receptors have the potential to produce long-lasting behavioural and neural alterations. The curcumin supplementation significantly modulates the altered gene expression of CREB in the cerebral cortex and cerebellum of diabetic rats to near control. In cerebral cortex insulin treatment doesn't show any significant effect in the CREB expression of diabetic rats whereas cerebellum shows a significant reversal. This study demonstrates that curcumin is having a modulatory effect in the transcription factor CREB expression which is crucial in maintaining the normal neuronal function and survival in diabetes. The dopamine D1 signal transduction pathway, activation of the transcription factor CREB, and dopamine-mediated gene expression are critically involved in memory processing, behavioural responses and drug addiction [63]. Interruption of this pathway can interfere with important cognitive performance and behavioural aspects associated with cerebral cortex and cerebellum. Dudman et al [64] reported that D2 receptors activate the cAMP response element-binding protein in neurons and D1 receptor stimulation leads to phosphorylation of the transcription factor Ca^2+ ^and CREB in the nucleus by means of NMDA receptor-mediated Ca ^2+ ^signaling. Thus we propose the importance of dopamine receptors in modulating CREB phosphorylation and activation. Possible interactions of other neurotransmitters with CREB is also suggested which needs further studies. The effect of curcumin in interacting with the dopaminergic receptor and CREB in STZ-induced diabetes proves its potential in managing CNS disorders in diabetes.

Phospholipase C mediates transduction of neurotransmitter signals across membranes via hydrolysis of phosphatidylinositol-4,5-bisphosphate, leading to generation of second messengers inositol- 1,4,5-trisphosphate and diacylglycerol. In the present study, we determined diabetes-mediated alterations in phospholipase C expression in the cerebral cortex and cerebellum. Further we extended the studies to phospholipase C regulation with curcumin supplementation and insulin treatment a potential therapeutic drug which can modulate signal transduction pathway there by contributing in the prevention of CNS dysfunction in diabetes. Our results showed a decreased expression of phospholipase C in the cerebral cortex and cerebellum of diabetic rats when compared to control. The DA D_1 _receptors show characteristic ability to stimulate adenylyl cyclase and generate inositol 1, 4, 5-trisphosphate (IP_3_) and diacylglycerol *via *the activation of phospholipase C [65,66]. We considered that the down regulation of the Phospholipase C in rat cerebral cortex and cerebellum during diabetes could contribute to the impaired signal transduction of G-protein coupled neurotransmitter receptors. Phospolipase C performs a catalytic mechanism, generating inositol triphosphate (IP_3_) and diacylglycerol (DAG). Altered phospholipase C expression fails to modulate the activity of downstream proteins important for cellular signalling. Defective expression of phospholipase C results in low levels of IP3 causing the impaired release of calcium and bring down the level of intracellular calcium and thus failed to execute the normal neuronal function in cerebral cortex and cerebellum. The previous study reports that phospholipase C-mediated signaling, initiated by growth factor receptor types, are involved in long-term memory formation, a process that requires gene expression [67]. Activation of all the G protein coupled receptors including Ach, glutamate and dopamine results in second messenger enzyme, phospholipase C expression. These evidences led us to propose that the enhancement of diabetes-mediated phospholipase C gene expression could impart damage to the central cognitive functions; which has been found to be effectively protected by curcumin treatment. Further studies are to be carried out to reveal the correlation between the expression of phospholipase C and G protein coupled neurotransmitter receptors.

The possible mechanism of curcumin action in CNS may be by lowering the blood glucose level which results in rendering the anti-apoptotic property [68]. Curcumin could reduce neuronal loss of the ischemic brain tissue, and inhibit expression of the activated caspase-3, a key executor of apoptosis [69,70]. Damage to neurons may occur through oxidative stress and/or mitochondrial impairment and culminate in activation of an apoptotic stage. Apoptosis or related phenomena are possibly involved in secondary cell death in diabetes. These results imply a potential therapeutic efficacy, i.e., curcumin may be used clinically as a neuroprotective drug for treatment of patients suffering from diabetes.

Insulin and sulfonylurea therapy for diabetes mellitus carries the risk of hypoglycaemic brain injury, and this risk is a major impediment to optimal glucose regulation in diabetic patients [71]. Factors that contribute to cognitive deficits as well as the protective factors that reduce the impact of diabetes on brain functions are still an enigma. Cerebral cortex and cerebellum are involved in cognitive, motor, and neuroendocrine activities [72-74]; thus, their affectations during diabetes are relevant in the pathogenesis of the disease. In addition, curcumin have recently received considerable attention since they have been shown to protect neurons against a variety of experimental neurodegenerative conditions. In the present investigation the generation of unique functional properties of curcumin via dopamine D1, D2 receptors, CREB and phospholoipase C interactions may yield a better understanding of behaviour and CNS disorders induced by diabetes.

## Abbreviations

STZ: Streptozotocin; CREB: Cyclic AMP response element binding protein; CNS: Central nervous system.

## Competing interests

The authors declare that they have no competing interests.

## Authors' contributions

TPK and CSP designed research. TPK, SA, GG and NG carried out the experiments and drafted manuscript. All authors read and approved the final manuscript.

## References

[B1] McCall Millington Wr Wurtman RJ Metabolic fuel and amino acid transport into the brain in experimental diabetes mellitus Proc Nadl Acad Sci USA 1982 97 2881 2885 10.1073/pnas.79.17.5406 PMC346906 6752947

[B2] Nagy R O' Connor A Kempers S yeo R Qualis C Adaption in brain glucose uptake following recurrent hypoglycaemia Proc Acad Sci USA 1994 91 9352 69356 10.1073/pnas.91.20.9352 PMC44810 7937768

[B3] Brownlee M Biochemistry and molecular cell biology of diabetic complications Nature 2001 414 813 820 10.1038/414813a 11742414

[B4] Pardridge WM Brain metabolism: a perspective from the blood-brain barrier Physiol Rev 1983 63 1481 1535 6361813 10.1152/physrev.1983.63.4.1481

[B5] Feldman EL Stevens MJ Greene DA Pathogenesis of diabetic neuropathy Clin Neurosci 1997 4 365 370 9358981

[B6] Auer RN Siesjo BK Hypoglycaemia: brain neurochemistry and neuropathology Baillieres Clin Endocrinol Metab 1993 7 611 625 10.1016/S0950-351X(05)80210-1 8379907

[B7] Kamal A Biessels GJ Duis SE Gispen WH Learning and hippocampal synaptic plasticity in streptozotocin-diabetic rats: interaction of diabetes and ageing Diabetologia 2000 43 500 506 10.1007/s001250051335 10819245

[B8] Ouyang L Wang J Zhu X Diagnostic efficacy of glutamic acid decarboxylase antibody and islet cell antibody in type I diabetes mellitus Zhonghua Nei Ke Za Zhi 2000 39 674 676 11374174

[B9] Osawa T Kato Y Protective role of antioxidative food factors in oxidative stress caused by hyperglycemia Ann N Y Acad Sci 2005 1043 440 451 10.1196/annals.1333.050 16037265

[B10] Ahmad M Turkseven S Mingone CJ Gupte SA Wolin MS Abraham NG Heme oxygenase-1 gene expression increases vascular relaxation and decreases inducible nitric oxide synthase in diabetic rats Cell Mol Biol (Noisy-le-grand) 2005 51 371 376 16309587

[B11] Ishrat T Khan MB Hoda MN Yousuf S Ahmad M Ansari MA Ahmad AS Islam F Coenzyme Q10 modulates cognitive impairment against intracerebroventricular injection of streptozotocin in rats Behav Brain Res 2006 171 9 16 10.1016/j.bbr.2006.03.009 16621054

[B12] Itokawa H Hirayama F Funakoshi K Takeya K Studies on the antitumor bisabolane sesquiterpenoids isolated from Curcuma xanthorrhiza Chem Pharm Bull 1985 33 3488 3492 4085078 10.1248/cpb.33.3488

[B13] Bala K Tripathy BC Sharma D Neuroprotective and anti-ageing effects of curcumin in aged rat brain regions Biogerontology 2006 7 81 9 10.1007/s10522-006-6495-x 16802111

[B14] Kuhad A Chopra K Curcumin attenuates diabetic encephalopathy in rats: behavioral and biochemical evidences Eur J Pharmacol 2007 576 34 42 10.1016/j.ejphar.2007.08.001 17822693

[B15] Shishodia S Sethi G Aggarwal BB Curcumin: getting back to the roots Ann N Y Acad Sci 2005 1056 206 217 10.1196/annals.1352.010 16387689

[B16] Marchall JF Friedman MI Heffner TG Reduced anorexic and locomotor-stimulant action of d-amphetamine in alloxan-diabetic rats Brain Res 1976 111 428 32 10.1016/0006-8993(76)90789-7 949615

[B17] Waxman SG Sabin TD Diabetic truncal polyneuropathy Arch Neurol 1981 38 46 7 7458723 10.1001/archneur.1981.00510010072013

[B18] Vallone D Picetti R Borrelli E Structure and function of dopamine receptors Neurosci Biobehav Rev 2000 24 125 132 10.1016/S0149-7634(99)00063-9 10654668

[B19] Nogueira CR Machado UF Curi R Carpinelli AR Modulation of insulin secretion and 45Ca2+ efflux by dopamine in glucose-stimulated pancreatic islets Gen Pharmacol 1994 25 909 16 7835636 10.1016/0306-3623(94)90095-7

[B20] Barik S de Beaurepaire R Evidence for a functional role of the dopamine D3 receptors in the cerebellum Brain Res 1996 737 1-2 347 350 10.1016/0006-8993(96)00964-X 8930390

[B21] Slater Lauren Opening Skinner's Box: Great Psychological Experiments of the Twentieth Century 2005 New York: W. W. Norton & Company 86 90

[B22] Spaulding SW The ways in which hormones change cyclic adenosine 3',5'-monophosphate-dependent protein kinase subunits, and how such changes affect cell behavior Endocr Rev 1993 14 5 632 650 8262010 10.1210/edrv-14-5-632

[B23] Shimomura A Okamoto Y Hirata Y Kobayashi M Kawakami K Kiuchi K Wakabayashi T Hagiwara M Dominant negative ATF1 blocks cyclic AMP-induced neurite outgrowth in PC12 D cells J Neurochem 1998 70 3 1029 34 9489722 10.1046/j.1471-4159.1998.70031029.x

[B24] Whiting PH Palmano KP Howthorne JN Enzymes of myoinositol and inositol lipid metabolism in rats with streptozotocin induced diabetes Biochem J 1979 179 549 553 224862 10.1042/bj1790549 PMC1186662

[B25] Junod A Lambert AE Staufferacher W Renold AE Diabetogenic action of Streptozotocin: Relationship of dose to metabolic response J Clin Invest 1969 48 2129 2139 10.1172/JCI106180 4241908 PMC297467

[B26] Hohenegger M Rudas B Kidney failure in experimental diabetes mellitus Wien Z Inn Med 1971 52 1 36 40 5540817

[B27] Arison RN Ciaccio EI Glitzer MS Cassaro JA Pruss MP Light and electron microscopy of lesions in rats rendered diabetic with streptozotocin Diabetes 1967 16 51 56 6015682 10.2337/diab.16.1.51

[B28] Sharma S Kulkarni SK Agrewala JN Chopra K Curcumin attenuates thermal hyperalgesia in a diabetic mouse model of neuropathic pain Eur J Pharmacol 2006 536 256 261 10.1016/j.ejphar.2006.03.006 16584726

[B29] Glowinski J Iversen LL Regional studies of catecholamines in the rat brain J Neurochem 1966 13 655 659 10.1111/j.1471-4159.1966.tb09873.x 5950056

[B30] Madras BK Fahey MA Canfield DR Spealman RD D1 and D2 dopamine receptors in caudate-putamen of nonhuman primates (Macaca fascicularis) J Neurochem 1988 51 934 943 10.1111/j.1471-4159.1988.tb01830.x 2970527

[B31] Lowry OH Rosenbrough NH Farr AL Randall RJ Protein measurement with folin Phenol reagent J Biol Chem 1951 193 265 275 14907713

[B32] Scatchard G The attraction of proteins for small molecules and ions Ann NY Acad Sci 1949 51 660 72 10.1111/j.1749-6632.1949.tb27297.x

[B33] Aragno M Parola S Brignardello E Mauro A Tamagno E Manti R Danni O Boccuzzi G Dehydroepiandrosterone prevents oxidative injury induced by transient ischemia/reperfusion in the brain of diabetic rats Diabetes 2000 49 1924 1931 10.2337/diabetes.49.11.1924 11078461

[B34] Low PA Nickander KK Tritschler HJ The role of oxidative stress and antioxidant treatment in experimental diabetic neuropathy Diabetes 1997 44 46:38 10.2337/diab.46.2.s38 9285497

[B35] Meghana K Sanjeev G Ramesh B Curcumin prevents streptozotocin-induced islet damage by scavenging free radicals: a prophylactic and protective role Eur J Pharmacol 2007 577 183 191 10.1016/j.ejphar.2007.09.002 17900558

[B36] Seo KI Choi MS Jung UJ Kim HJ Yeo J Jeon SM Lee MK Effect of curcumin supplementation on blood glucose, plasma insulin, and glucose homeostasis related enzyme activities in diabetic db/db mice Mol Nutr Food Res 2008 52 995 1004 10.1002/mnfr.200700184 18398869

[B37] McCall AL The impact of diabetes on the CNS Diabetes 1992 41 557 570 10.2337/diabetes.41.5.557 1568525

[B38] Missale C Nash SR Robinson SW Jaber MC Dopamine receptors: From structure to function Physiol Rev 1998 78 189 225 9457173 10.1152/physrev.1998.78.1.189

[B39] Lozovsky D Saller CF Kopin IJ Dopamine receptor binding is increased in diabetic rats Science 1981 214 1031 1033 10.1126/science.6458088 6458088

[B40] Garris Age diabetes associated alterations in regional brain norepinephrine concentrations and adrenergic populations in C57BL/KsL mice Developmental Brain Research 1990 51 161 166 10.1016/0165-3806(90)90272-Z 2323025

[B41] Fink JS Smith GP Decreased locomotor and investigatory exploration after denervation of catecholamine terminal fields in the forebrain of rats J Comp Physiol Psychol 1979 93 34 65 10.1037/h0077587 447888

[B42] Funada M. Suzuki T Misawa M The role of dopamine D1-receptor in morphine induced hyperlocomotion in mice Neurosci Lett 1994 169 1 4 10.1016/0304-3940(94)90342-5 7914011

[B43] Marshall JF Friedman MI Heffner TG Reduced anorexic and locomotor-stimulant action of D-amphetamine in alloxan-diabetic rats Brain Res 1976 111 428 432 10.1016/0006-8993(76)90789-7 949615

[B44] Bhattacharya SK Saraswathi M Effect of intracerebroventricularly administered insulin on brain monoamines and acetylcholine in euglycemic and alloxan- induced hyperglycemic rats Indian J Exp Biol 1991 29 1095 1100 1816091

[B45] Lackovic Z Salkovic M Kuci Z Relja M Effect of long-lasting diabetes mellitus on rat and human brain monoamines J Neurochem 1990 51 143 147 10.1111/j.1471-4159.1990.tb13294.x 2293606

[B46] Kamei J Saitoh A Iwamoto Y Funada M Suzuki T Misawa M Nagase H Kasuya Y Effects of diabetes on spontaneous locomotor activity in mice Neurosci Lett 1994 178 69 72 10.1016/0304-3940(94)90292-5 7816344

[B47] de Paulis T The discovery of epidepride and its analogs as highaffinity radioligands for imaging extrastriatal dopamine D2 receptors in human brain Curr Pharm Des 2003 9 673 696 10.2174/1381612033391135 12570798

[B48] Verma A Moghaddam B NMDA receptor antagonists impair prefrontal cortex function as assessed via spatial delayed alternation performance in rats: modulation by dopamine J Neurosci 1996 16 373 379 8613804 10.1523/JNEUROSCI.16-01-00373.1996 PMC6578732

[B49] Castellano C Ventura R Cabib S Puglisi-Allegra S Strain-dependent effects of anandamide on memory consolidation in mice are antagonized by naltrexone Behav Pharmacol 1999 10 453 457 10.1097/00008877-199909000-00003 10780251

[B50] Zhou QY Palmiter RD Dopamine-deficient mice are severely hypoactive, adipsic, and aphagic Cell 1995 83 1197 1209 10.1016/0092-8674(95)90145-0 8548806

[B51] Kobayashi M Shigeta Y Anti-insulin receptor antibody--its measurement and significance Nippon Rinsho 1990 48 308 14 1707991

[B52] Shimomura YSH Takahashi M Uehara Y Kobayashi I Kobayashi S Ambulatory activity and dopamine turnover in streptozotocin-induced diabetic rats Exp Clin Endocrinol 1990 95 385 388 10.1055/s-0029-1210980 2147142

[B53] Kolasiewicz W Maj J Locomotor hypoactivity and motor disturbances-behavioral effects induced by intracerebellar microinjections of dopaminergic DA-D2/D3 receptor agonists Pol J Pharmacol 2001 53 509 15 11990070

[B54] Gireesh G Balarama Kaimal S Peeyush Kumar T Paulose CS Decreased muscarinic M1 receptor gene expression in the hypothalamus, brainstem, and pancreatic islets of streptozotocin-induced diabetic rats Journal of Neuroscience Research 2008 86 947 953 10.1002/jnr.21544 17960828

[B55] Mohanan VV Kaimal SB Paulose CS Decreased 5-HT1A receptor gene expression and 5HT1A receptor protein in the cerebral cortex and brain stem during pancreatic regeneration in rats Neurochemical Research 2005 30 25 32 10.1007/s11064-004-9682-7 15756929

[B56] Kaimal SB George KA Paulose CS Gamma-aminobutyric acid A receptor functional decrease in the hypothalamus during pancreatic regeneration in rats Pancreas 2008 37 e20 30 10.1097/MPA.0b013e3181661af4 18580435

[B57] Anu J Peeyush Kumar T Nandhu MS Paulose CS Enhanced NMDAR1, NMDA2B and mGlu5 receptors gene expression in the cerebellum of insulin induced hypoglycaemic and streptozotocin induced diabetic rats Eur J Pharmacol 2010 630 61 68 10.1016/j.ejphar.2009.12.024 20056114

[B58] Puglisi-Allegra S Cestari V Cabib S Castellano C Strain-dependent effects of post-training cocaine or nomifensine on memory storage involve both D1 and D2 dopamine receptors Psychopharmacology 1994 115 157 162 10.1007/BF02244766 7862889

[B59] Imperato A Obinu MC Gessa GL Stimulation of both dopamine D1 and D2 receptors facilitates in vivo acetylcholine release in the hippocampus Brain Research 1993 618 341 345 10.1016/0006-8993(93)91288-4 8104086

[B60] Umegaki H Munoz J Meyer RC Spangler EL Yoshimura J Ikari H Iguchi A Ingram DK Involvement of dopamine D(2) receptors in complex maze learning and acetylcholine release in ventral hippocampus of rats Neuroscience 2001 103 27 33 10.1016/S0306-4522(00)00542-X 11311785

[B61] Finkbeiner S CREB couples neurotrophin signals to survival messages Neuron 2000 25 11 14 10.1016/S0896-6273(00)80866-1 10707967

[B62] Mayr B Montminy M Transcriptional regulation by the phosphorylation-dependent factor CREB Nat Rev Mol Cell Biol 2001 2 599 609 10.1038/35085068 11483993

[B63] Nestler EJ. Total recall-the memory of addiction Neurobiology Science 2001 292 2266 2267 10.1126/science.1063024 11423644

[B64] Dudman Joshua JT Eaton Molly E Rajadhyaksha Anjali Taher Wendy Mac Muffadal Barczak Amy Kameyama Kimihiko Huganir Richard Konradi Christine Dopamine D1 receptors mediate CREB phosphorylation via phosphorylation of the NMDA receptor at Ser897-NR1 J Neurochem 2003 87 922 934 10.1046/j.1471-4159.2003.02067.x 14622123 PMC4203348

[B65] Monsma F Mahan L McVittie L Gerfen C Sibley D Molecular cloning and expression of a D1 dopamine receptor linked to adenylyl cyclase activation Proc Natl Acad Sci 1990 87 6723 6727 10.1073/pnas.87.17.6723 2168556 PMC54609

[B66] Sibley DR Monsma FJ Jr Shen Y Molecular neurobiology of dopaminergic receptors Int Rev Neurobiol 1993 35 391 415 10.1016/S0074-7742(08)60573-5 8463063

[B67] Orbana Paul C Paul FC Riccardo B Is the Ras-MAPK signalling pathway necessary for long-term memory formation? Trends in Neurosciences 1999 22 38 44 10.1016/S0166-2236(98)01306-X 10088998

[B68] Zhao Jing Zhao Yong Zheng Weiping Lu Yuyu Feng Gang Yu Shanshan Neuroprotective effect of curcumin on transient focal cerebral ischemia in rats Brain Research 2008 1229 224 232 10.1016/j.brainres.2008.06.117 18640105

[B69] Ashe PC Berry MD Apoptotic signaling cascades. Prog. Neuro-psychopharmacol Biol Psychiatry 2003 27 199 214 10.1016/S0278-5846(03)00016-2 12657360

[B70] Guan QH Pei DS Liu XM Wang XT Xu TL Zhang GY Neuroprotection against ischemic brain injury by SP600125 via suppressing the extrinsic and intrinsic pathways of apoptosis Brain Res 2006 1092 36 46 10.1016/j.brainres.2006.03.086 16674927

[B71] Davis EA Keating B Byrne GC Russell M Jones TW Impact of improved glycaemic control on rates of hypoglycaemia in insulin dependent diabetes mellitus Arch Dis Child 1998 78 111 115 10.1136/adc.78.2.111 9579150 PMC1717459

[B72] Dube MG Torto R Kalra SP Increased leptin expression selectively in the hypothalamus suppresses inflammatory markers CRP and IL-6 in leptin-deficient diabetic obese mice Peptides 2008 29 593 598 10.1016/j.peptides.2008.01.001 18325632 PMC2291149

[B73] Gao Q Horvath TL "Cross-talk between estrogen and leptin signaling in the hypothalamus," American Journal of Physiology 2008 294 E817 E826 18334610 10.1152/ajpendo.00733.2007

[B74] Gerozissis K "Brain insulin, energy and glucose homeostasis; genes, environment and metabolic pathologies," European Journal of Pharmacology 2008 585 38 49 10.1016/j.ejphar.2008.01.050 18407262

